# Association between the size of healthcare facilities and the intensity of hypertension therapy: a cross-sectional comparison of prescription data from insurance claims data

**DOI:** 10.1038/s41440-020-00549-2

**Published:** 2020-09-15

**Authors:** Shusuke Hiragi, Noriaki Sato, Eiichiro Uchino, Tomohiro Kuroda, Motoko Yanagita

**Affiliations:** 1 Department of Nephrology, Graduate School of Medicine, Kyoto University, 54 Kawaharacho, Shogoin, Sakyo-ku, Kyoto 606-8507 Japan; 2 Division of Medical Informatics and Administration Planning, Kyoto University Hospital, 54 Kawaharacho, Shogoin, Sakyo-ku, Kyoto 606-8507 Japan; 3 Department of Biomedical Data Intelligence, Graduate School of Medicine, Kyoto University, 54 Kawaharacho, Shogoin, Sakyo-ku, Kyoto 606-8507 Japan; 4 Institute for the Advanced Study of Human Biology (ASHBi), Kyoto University, Yoshida-Konoe-cho, Sakyo-ku, Kyoto 606-8501 Japan

**Keywords:** Antihypertensive agents, Hypertension, Administrative claim

## Abstract

Hypertension is a heterogeneous disease for which role sharing in treatment between specialized facilities and small clinics is needed for efficient healthcare provision. However, the Japanese healthcare system has a “free access” attribute; therefore, nobody can control treatment resource allocation. We aimed to describe the current situation of role sharing by comparing antihypertensive therapies among different types of medical facilities. We analyzed 1% sampled Japanese medical insurance claims data related to outpatient care as of October 2014. We divided the target patients into four groups according to the size of the facilities that issued the insurance claim for them. Among these groups, we compared the number of antihypertensive drugs and proportion of difficult-to-treat hypertensive cases and performed a stratified analysis. The proportion of patients with hypertension and diabetes mellitus receiving renin-angiotensin-aldosterone system inhibitors (RAASis) as the first-choice drug was also compared. We identified 3465, 1797, 2323, and 34,734 claims issued from large, medium-sized, small hospitals, and clinics, respectively. The mean number of hypertensive drugs was 1.96, 1.87, 1.81, and 1.69, respectively, and the proportion of difficult-to-treat hypertensive cases was 18.9, 17.0, 14.3, and 12.0%, respectively, with both showing significant differences. Stratified analysis showed similar results. The proportion of patients with hypertension and diabetes mellitus receiving RAASis as the first-choice drug was higher in large hospitals than in clinics. In conclusion, facility size is positively associated with the number of antihypertensive drugs and proportions of difficult-to-treat hypertensive cases. This finding describes the current role sharing situation of hypertension therapy in the Japanese healthcare system with a “free-access” attribute.

## Introduction

Hypertension is a common disease that can ultimately result in diseases causing a poor quality of life, such as cardiovascular complications [1]. Hence, controlling the disease has an important meaning to our society’s productivity and the welfare of its members. Most patients with hypertension can be treated well with a small number of antihypertensive drugs, but a portion of them have refractory hypertension, which is defined as uncontrolled blood pressure despite the use of ≥3 antihypertensive agents, including diuretics [2], and the other portion of these patients also has an increased risk due to the combination of other lifestyle-related diseases, such as diabetes mellitus [3] or dyslipidemia [4].

Numerous studies have proven the positive relationship between hospital size and patient outcome in general. In particular, outcomes for specific surgeries, such as coronary artery bypass grafts, are famous for the relationship between outcome and the size of the hospital [5, 6]. Moreover, a small retrospective study reported their relationship in the field of hypertension [7]. The size is said to be important due to the accumulation of patients, but treating all patients with hypertension at these facilities is impossible due to limited resources.

Therefore, distinguishing high-risk and low-risk patients and allocating appropriate institutions for them are important. The healthcare system in Japan has a “free access” attribute [8], in which every citizen can select any healthcare institution from small clinics to the largest hospitals freely, so that no healthcare professional, insurer, or government agency can manage patient allocation for appropriate facilities considering their severity, in contrast to many countries in the world where general practitioners play the role of gatekeeper [9]. For example, common forms of healthcare insurance in the United States require referrals from primary care physicians (PCPs) or higher copayments for a specialist consultation. In other countries, such as the United Kingdom and Italy, visits for specialists require a referral from PCPs, while visiting a specialist without a referral results in financial penalties in France [10]. Previous studies [11, 12] have suggested an association between health expenditure and the gatekeeping function, partially through effective resource allocation.

In such a situation, the extent of role sharing in hypertension treatment between large-scale facilities and small clinics in Japan is scarcely known because of the limited data until now, when the national health insurance claims data have become accessible to researchers. In the present study, we aimed to examine the association between the intensity of antihypertensive therapy and the size of healthcare facilities to describe the current situation of role sharing in hypertension therapy in the country by analyzing the healthcare insurance claims database in Japan, which covers almost all of the citizens in the country. We also examined the association between the guideline adherence rate in a specific condition, patients with diabetes mellitus, and the size of facilities to reinforce the aforementioned intention.

## Methods

### Study design

This was a cross-sectional study comparing hypertension treatment with the size of facilities treating patients by using health insurance claims data in Japan. We identified people who had prescriptions including at least one antihypertensive drug in October 2014 from the 1% sampled claims data related to outpatient care. We divided these potentially eligible patients into four groups according to the size of the prescribing facilities and compared the number of antihypertensive drugs and the proportions of patients treated with ≥3 classes of antihypertensive drugs among these groups.

### Data source

We utilized the health insurance claims database in Japan provided by the Ministry of Health, Labor, and Welfare (MHLW). Japan has a universal health insurance system covering almost all of its citizens [13]. Although more than 3000 health insurers exist [14], the medical fee scale is uniquely determined by government agencies, and most healthcare facilities claim reimbursement within the scale. The reimbursement claim from healthcare facilities to insurers is sent electronically, and the format for claims is also uniquely determined in the country. The Japanese Act on Assurance of Medical Care for Elderly People requires MHLW to collect reimbursement claims from all over the country to improve the healthcare provision system. Based on the act, MHLW has developed and managed a database of insurance claims [15]. We requested permission to utilize the database for MHLW based on the aforementioned act, which also requests MHLW to provide a portion of the database to researchers for the purpose of public health research since 2011 [16]. Our request was approved, and the data were provided with adequate sampling and anonymization.

Adhering to the regulation, MHLW provided us with one-month of sampled data as of October 2014, which were related to outpatient care. The data were a 1% sampled dataset from the aforementioned outpatient care data, with matched age and sex distributions to the original data. For anonymization, patients’ name and ID were removed, age was aggregated into 5-year tiers, and personal address and information (e.g., names and addresses) of the facilities issuing the claim were masked.

### Inclusion and exclusion criteria

From the sampled insurance claims data, we included patients who were prescribed at least one hypertensive drug for >27 days, with the intent to analyze hypertension therapy in a stable state. In contrast, those claims containing only prescriptions of <28 days for each antihypertensive drug were excluded.

### Definition of exposure

In the present study, we set the difference in the size of medical facilities as the exposure. In particular, we divided the insurance claims data into the following four groups according to the issuing facilities: large hospitals (defined as equipped with ≥200 beds), medium-sized hospitals (100 ≤ beds < 200), small hospitals (20 ≤ beds < 100), and clinics (equipped with no or <20 beds). A clinic was defined according to the classification, described in the Japanese Medical Care Act. The distinction of each size of hospitals and clinics was based on the type of specific insurance claims issued by them. In particular, only large hospitals were eligible to charge the “outpatient examination fee”; therefore, we labeled claims containing the fee as issued by large hospitals. In contrast, the “re-examination fee” is only eligible for facilities that were equipped with <200 beds or clinics. We extracted the claims containing the “re-examination fee” followed by dividing them with claim codes of “specific disease follow-up management fee”, to which the Japanese medical fee scale allocates different codes according to the size of the claiming facility (medium-sized hospital, small hospital, and clinics). Those claims containing the aforementioned claim codes were regarded as issued by each type of facility.

We excluded claims that did not contain “outpatient examination fee” or “re-examination fee” as well as those containing “re-examination fee” but not “specific disease follow-up management fee”.

In fact, the original dataset that MHLW manages for administrative purposes includes the names of medical facilities, but ministerial regulation did not allow academic researchers to utilize this data, and MHLW masked them before they provided data to researchers. Therefore, we used the aforementioned method to distinguish the size of facilities.

### Definition of outcome

#### Antihypertensive therapy

As a primary analysis, we counted the number of antihypertensive drugs contained in each insurance claim. Every claim was issued once in a month for each patient; therefore, the counted data included all the antihypertensive drugs prescribed in the month. We defined antihypertensive drugs as ATC codes of C02, C03, C04, C07, C08, and C09. For combination agents, we counted them as if patients took the component drugs separately. The classification of drugs was based on The Japanese Society of Hypertension Guidelines for the Management of Hypertension [17] and a textbook of therapeutic drugs published in Japan [18].

In addition, we compared the proportion of patients with difficult-to-treat hypertension, who were defined as patients prescribed ≥3 classes of antihypertensive drugs, considering the definition of refractory hypertension; given that we could not extract refractory hypertension patients from the claims data only, we used the aforementioned definition. The classes of antihypertensive drugs were set as follows: renin-angiotensin-aldosterone system inhibitors (RAASis, including angiotensin-converting enzyme inhibitors, angiotensin II receptor blockers, and direct renin inhibitors), calcium channel blockers, beta blockers, thiazides, diuretics, and others.

### Stratified analysis

Our main interests were to compare the hypertension treatment among the facilities with different sizes. However, relatively large facilities tended to receive more difficult-to-treat patients; thus, confounders related to demographic characteristics and comorbidities may exist. In the present study, we stratified patients’ age, coexistence of diabetes mellitus, coexistence of dyslipidemia, and coexistence of kidney disease to control the aforementioned potential confounders as well as showing the crude results. In particular, we divided the claims according to the patients’ age as ≥75 years or not, considering the definition of the latter-stage elderly in Japan. For diabetes mellitus, we extracted claims including oral hypoglycemic agents or insulins equivalent to ATC codes of A10. Similarly, we defined patients with dyslipidemia as those with prescription of hypolipidemic agents, equivalent to ATC codes of C10. Regarding the coexistence of kidney disease, we utilized diagnosis information equivalent to ICD-10 codes of N00–N08 (glomerular diseases), N10–N16 (renal tubule-interstitial diseases), N17–N19 (acute kidney failure and chronic kidney disease), and N25–N29 (other disorders of kidney and ureter).

### RAASis in patients with diabetes mellitus

Among patients with hypertension, the recommended treatment for those with accompanying diabetes mellitus is different from that of other patients [19]. In particular, RAASis, including angiotensin-converting-enzyme inhibitors and angiotensin II receptor blockers, were designated as having a positive indication to patients with hypertension and diabetes mellitus in the latest Japanese guideline for the management of hypertension available at the time when our data were issued as insurance claims [17]. Here, we compared the proportion of adherence to the aforementioned recommendation among facility sizes by extracting claims with only one type of antihypertensive agent used and dividing them by the coexistence of diabetes mellitus, followed by calculating the proportion of RAASi used among them. The coexistence of diabetes mellitus was recognized when the claim concurrently included the prescription of oral hypoglycemic agents or insulins equivalent to ATC codes of A10, similar to that mentioned in the above section. RAASi was defined as ATC codes of C09.

### Statistical analysis

We utilized the Kruskal–Wallis test on the four groups (large, medium-sized, and small hospitals and clinics) to evaluate the existence of a significant difference in the average of the continuous values, such as age or number of antihypertensive drugs. When statistical significance was detected, we performed ad hoc Welch’s *t*-test to evaluate the significant difference between each group of hospitals and clinics. For the proportions, we utilized Chi-square tests to evaluate the differences among the four groups and one-to-one comparisons between each hospital group and clinics. We set the 5% threshold as representing statistical significance. Age and sex distribution adjustment was performed by the direct method using the clinics group as a reference population, reflecting differences in those demographics in each group discussed below. R Version 3.2.1 software [20] was used for all analyses.

## Results

**Fig. 1:**
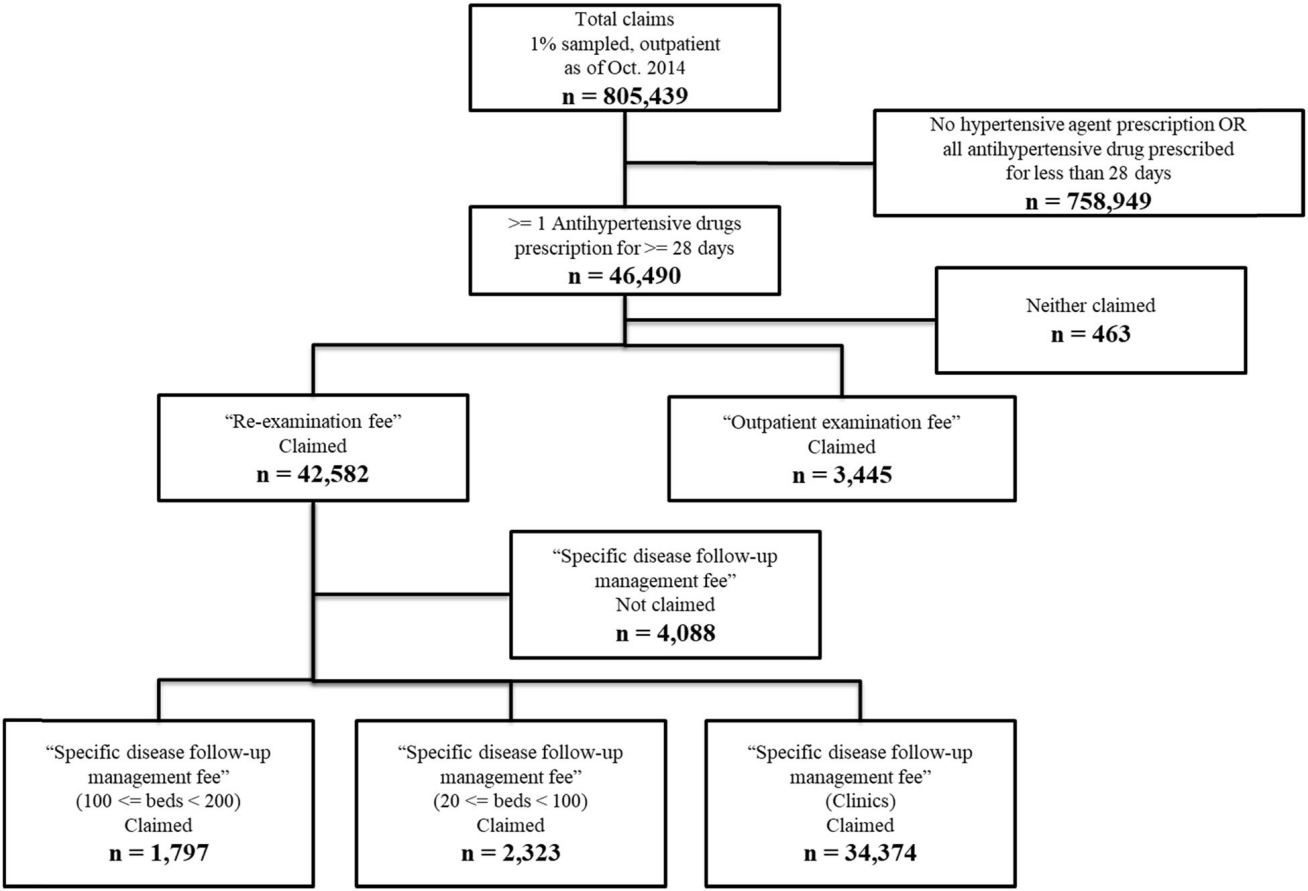
Inclusion and group flow diagram of the target populations. According to Japanese regulation, facilities equipped with <20 beds are classified as clinics

From a total of 805,439 claims contained in a sampled database, 46,690 claims included at least one antihypertensive drug prescribed for ≥28 days. Among them, 3445 contained the “outpatient examination fee” and were categorized as large hospital-issued claims. Similarly, 1797 were categorized as medium-sized hospital-issued claims, 2323 as small hospital-issued claims, and 34,734 as clinic-issued claims (Fig. 1). That is, approximately 80% of patients with hypertension were followed-up in small clinics.

The patients’ characteristics are shown in Table 1. The mean patient age was slightly >70 years in all groups. The proportion of patients with diabetes mellitus, dyslipidemia, and kidney disease was higher in large facilities than in smaller facilities, but the difference between medium-sized/small-sized hospitals and clinics was relatively small. Age and sex distribution showed statistical significance in each group; hence, we performed adjustments, as mentioned above.

**Table 1:** Characteristics of the included patients (*n* = 41,939)

Characteristics	Large hospitals *n* = 3445	*p* (comparator:clinics)	Medium-sized hospitals *n* = 1797	*p* (comparator:clinics)	Small hospitals *n* = 2323	*p* (comparator: clinics)	Clinics *n* = 34,374	*p* (all groups)
Age, mean	70.56	0.002	72.94	<0.001	73.15	<0.001	71.27	<0.001
Female gender	45.9%	<0.001	52.0%	0.03	53.0%	0.15	54.6%	<0.001
Co-existence of diabetes mellitus	23.3%	<0.001	19.5%	<0.001	16.6%	<0.001	13.2%	<0.001
Co-existence of dyslipidemia	41.9%	<0.001	39.1%	0.33	39.1%	0.27	38.0%	<0.001
Concurrent diagnosis related to chronic kidney disease	16.2%	<0.001	8.2%	<0.001	7.2%	<0.001	4.8%	<0.001

**Fig. 2:**
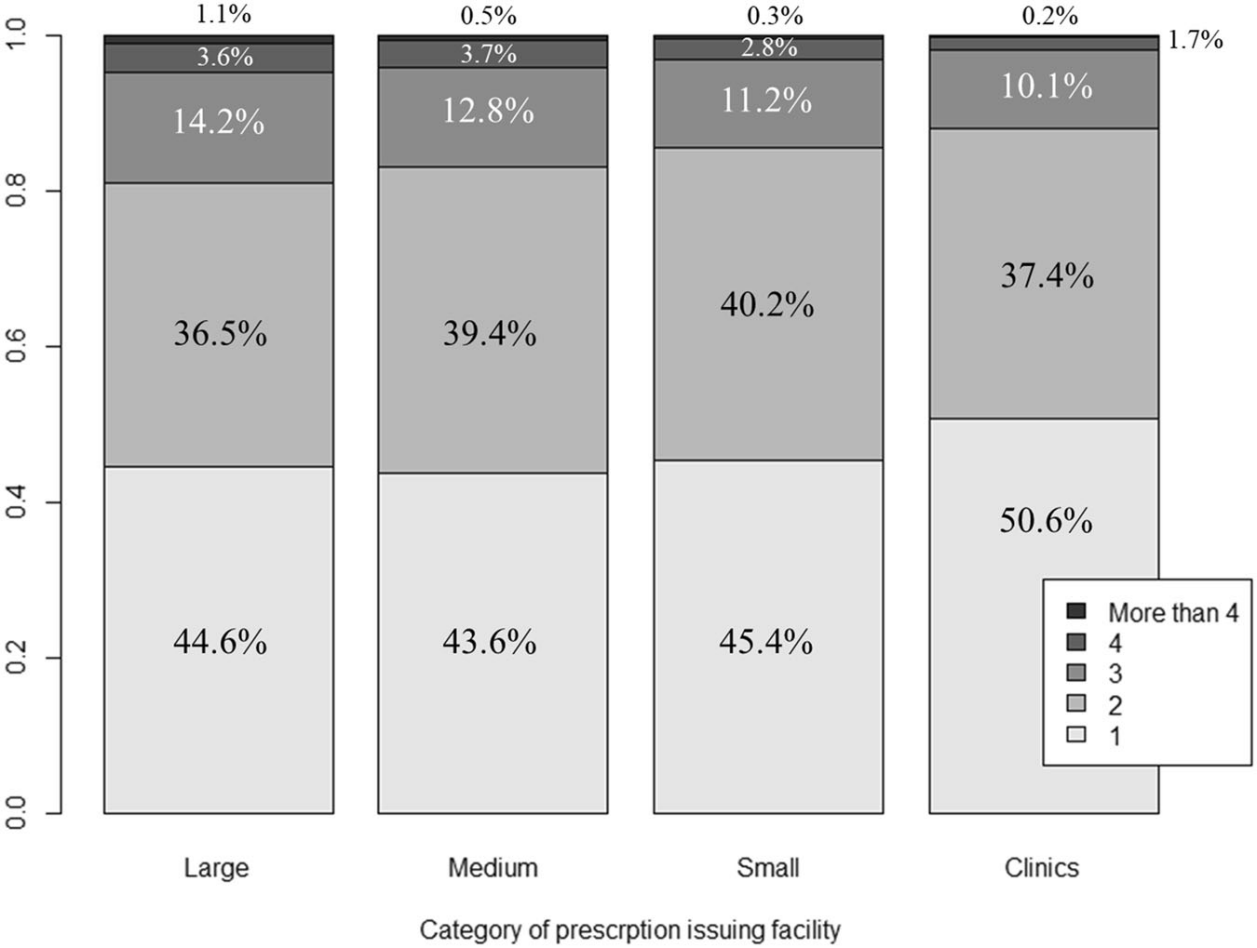
Number of types of antihypertensive drugs prescribed and their proportions

Regarding the main analysis, the mean numbers of hypertensive drugs were 1.96, 1,87, 1.81, and 1.69 in large facilities, medium-sized hospitals, small-sized hospitals, and clinics, respectively (Table 2). The proportion of patients prescribed >2 classes of antihypertensive drugs or diuretics was 18.9, 17.0, 14.3, and 12.0% in large-sized, medium-sized, and small-sized hospitals and clinics, respectively. These differences were statistically significant. The proportions of patients, including the number of each type of antihypertensive drug, are shown in Fig. 2. The results of age and sex adjustment are shown in a supplementary table and figure, and they did not show remarkable differences compared to the crude results.

**Table 2:** The number of antihypertensive drugs and the proportions of patients prescribed ≥3 classes of antihypertensive drugs in each group

Characteristics	Large hospitals	*p* (comparator:clinics)	Medium-sized hospitals	*p* (comparator:clinics)	Small hospitals	*p* (comparator:clinics)	Clinics	*p* (all groups)
Number of antihypertensive drugs, mean	1.96	<0.001	1.87	<0.001	1.81	<0.001	1.69	<0.001
Number of antihypertensive drugs, standard deviation	1.10		0.96		0.91		0.83	
Proportion of patients prescribed more than two classes of antihypertensive drugs	18.9%	<0.001	17.0%	<0.001	14.3%	<0.001	12.0%	<0.001

When we stratified the patients according to their age and the coexistence of diabetes mellitus and dyslipidemia, the mean number of antihypertensive drugs also increased with increasing facility size (Table 3), and the same tendency existed in the kidney disease stratum.

**Table 3:** The number of antihypertensive drugs prescribed in each stratified patient group

	Large hospitals				Medium-sized hospitals				Small hospitals				Clinics			*p* (all groups)	
Subgroups	*n*	Number of drugs, mean	Standard deviation	*p* (comparator:clinics)	*n*	Number of drugs, mean	Standard deviation	*p* (comparator: clinics)	*n*	Number of drugs, mean	Standard deviation	*p* (comparator:clinics)	*n*	Number of drugs, mean	Standard deviation	*n* (total)	*p*
Age ≥ 75	1479	2.01	1.11	<0.001	869	1.91	1.00	<0.001	1125	1.89	<0.001	0.98	14,162	1.74	0.88	17,635	<0.001
Age <75	1966	1.92	1.10	<0.001	928	1.83	0.92	<0.001	1198	1.74	<0.001	0.83	20,212	1.66	0.80	24,304	<0.001
Co-existence of diabetes mellitus	804	2.16	1.10	<0.001	351	2.03	0.96	<0.001	385	1.90	0.06	0.91	4529	1.81	0.83	6069	<0.001
Without diabetes mellitus	2641	1.90	1.06	<0.001	1446	1.83	0.94	<0.001	1938	1.79	<0.001	0.91	29,845	1.67	0.82	35,870	<0.001
Co-existence of dyslipidemia	1445	2.07	1.15	<0.001	703	1.89	0.99	<0.001	909	1.82	0.001	0.91	13,056	1.72	0.85	16,113	<0.001
Without dyslipidemia	2000	1.88	1.06	<0.001	1094	1.86	0.95	<0.001	1414	1.80	<0.001	0.91	21,318	1.68	0.82	25,826	<0.001
Co-existence of kidney disease	557	2.22	1.37	<0.001	147	2.17	1.17	0.04	167	2.11	0.11	1.23	1636	1.96	1.11	2,507	<0.001
Without kidney disease	2888	1.91	1.04	<0.001	1650	1.84	0.94	<0.001	2156	1.79	<0.001	0.88	32,738	1.68	0.82	39,432	<0.001

The proportion of patients prescribed >2 classes of antihypertensive drugs was also higher in larger facilities in each stratum, but the difference was relatively small, especially between small hospitals and clinics (Table 4). In each stratum, age and sex adjustment also made no remarkable difference (Supplementary Table).

**Table 4:** The proportions of patients prescribed ≥3 classes of antihypertensive drugs in each stratified patient group

	Large hospitals			Medium-sized hospitals			Small hospitals			Clinics		*p* (all groups)	
Subgroups	*n*	Proportion (%)	*p* (comparator:clinics)	*n*	Proportion (%)	*p* (comparator:clinics)	*n*	Proportion (%)	*p* (comparator:clinics)	*n*	Proportion (%)	*n* (total)	*p*
Age ≥ 75	1479	21.3	<0.001	869	18.6	<0.001	1125	15.8	0.05	14,162	13.7	17,635	<0.001
Age < 75	1966	17.1	<0.001	928	15.4	<0.001	1198	12.9	0.19	20,212	10.7	24,304	<0.001
Co-existence of diabetes mellitus	804	22.1	<0.001	351	21.9	0.001	385	17.4	0.3	4,529	15.3	6069	<0.001
Without diabetes mellitus	2641	17.9	<0.001	1,446	15.8	<0.001	1938	13.7	0.003	29,845	11.5	35,870	<0.001
Co-existence of dyslipidemia	1445	22.8	<0.001	703	18.1	<0.001	909	15.4	0.04	13,056	13.0	16,113	<0.001
Without dyslipidemia	2000	16.1	<0.001	1094	16.3	<0.001	1414	13.7	0.01	21,318	11.3	25,826	<0.001
Co-existence of kidney disease	557	26.2	0.008	147	31.3	0.004	167	25.1	0.22	1,636	20.7	2507	0.003
Without kidney disease	2888	17.5	<0.001	1650	15.7	<0.001	2156	13.5	0.006	32,738	11.5	39,432	<0.001

Table 5 shows the proportion of patients who were prescribed a RAASi as the first-choice drug among those with diabetes mellitus and those without diabetes mellitus. In the patients with diabetes mellitus, approximately 40–60% were prescribed a RAASi, and the proportion was larger in large hospitals and small hospitals than in clinics and medium-sized hospitals. In contrast, in patients without diabetes mellitus, the proportion was approximately 35%, and the difference among the groups was small, even though statistical significance was detected between small hospitals and clinics. Additionally, in this analysis, age and sex adjustment made no remarkable change in the crude result.

**Table 5:** The proportion of patients prescribed renin-angiotensin-aldosterone inhibitors as the first-choice drug in diabetes mellitus patients and those without the disease

	*n*	Large hospitals	*p* (comparator:clinics)	Medium-sized hospitals	*p* (comparator:clinics)	Small hospitals	*p* (comparator:clinics)	Clinics (%)	*p* (all groups)
Patients with diabetes (age and sex adjusted in parenthesis; reference group = clinics)	2480	60.2% (58.0%)	<0.001	42.9% (42.1%)	0.74	51.0% (47.4%)	0.02	41.0	<0.001
Patients without diabetes (age and sex adjusted in parenthesis; reference group = clinics)	17,700	32.8% (32.2%)	0.74	35.1% (35.6%)	0.14	37.6% (39.1%)	0.001	32.3	0.005

## Discussion

This study investigated the treatment differences among types of healthcare facilities. Our results showed that the number of types of antihypertensive drugs was larger in hospitals equipped with ≥200 beds than in smaller facilities, and the proportion of patients who were prescribed >2 antihypertensive drugs was the same. The difference showed statistical significance, indicating the association between facility size and the intensity of hypertension therapy. This result might indicate that the patients with difficult-to-control hypertension, to some extent, visit larger, experienced facilities, but the difference was not so large (1.96 drugs in large hospitals vs. 1.69 drugs in clinics).

A previous survey [21] estimated that the number of patients with hypertension in Japan is approximately 10 million. Our results extracted approximately 45,000 claims data, including at least one antihypertensive drug prescription from the 1% sampled database, indicating that approximately 4.5 million patients came to healthcare facilities in October 2014. Considering the follow-up interval, which even for white coat hypertension, is recommended to be 3–6 months [22], the current result seems to adequately reflect the real situation. In addition, a survey from a small cohort showed that 70.2% of patients go to clinics [23]. The result is similar to our current result, indicating that the external validity of our result is secured. Hence, our result can also be seen as capturing real-world data, making the data usable for understanding the current situation of hypertension treatment in Japan.

The clinical guidelines for hypertension recommend [2] that patients with resistant hypertension, defined as high blood pressure despite concurrent use of three antihypertensive agents of different classes including diuretics, should be referred to a hypertension specialist, who can help the patient achieve improved blood pressure control [7]. In addition, the assessment for secondary hypertension is recommended in the diagnostic algorithm shown in the aforementioned guidelines. The assessment includes specific imaging tests, including ultrasonography, computed tomography, magnetic resonance imaging, and renal angiography, which are more likely to be available in larger facilities than in small facilities. Considering our results showing that the proportion of patients prescribed >2 classes of antihypertensive drugs is almost 1.5 times larger in large-sized facilities than in clinics, a small portion of these patients were more likely to be treated and managed under adequate healthcare provision resources.

When we stratified the target population by age, coexistence with diabetes, and coexistence with dyslipidemia, the number of antihypertensive agents was also associated with the size of the prescribing facility. This result reinforced our primary result by showing that even if we excluded potential confounding factors, the aforementioned association exists. Regarding kidney disease, the same tendency was observed. However, the proportion of patients with concurrent diagnosis was much lower than 10%, which is a generally accepted prevalence of kidney diseases in Japan [24]. This discrepancy may be related to the low sensitivity of diagnosis information in claims data. This is due to a widely acknowledged practice in Japan that clinicians and administrative staff do not record diagnosis codes if those codes are not needed for reimbursement for specific examination or prescription, as also previously reported in Medicare claims data [25]. Hence, the current results related to kidney disease strata yielded limited information.

One possible interpretation for the aforementioned association is that spontaneous role sharing in healthcare facilities emerged even though the Japanese healthcare system has a “free-access” attribute (even though a special fee can be required when patients visit large hospitals without referral, the proportion of hospitals charging the fee was below 50% at that time [26], and the healthcare provision system in Japan was still regarded as having a “free-access” attribute [27, 28]). Patients with refractory conditions could be referred from small, general practitioner-like facilities to large-scale facilities to seek more specialized treatment, which results in the aforementioned association. However, the difference in the proportion of patients with >2 antihypertensive drugs was not so large, indicating that more role sharing can be achieved for our society. At least, we succeeded in showing the current status of hypertension therapy in the present study for future healthcare policy making.

Diabetes mellitus is frequently accompanied by hypertension [29], mainly because diabetes mellitus may affect the occurrence of hypertension, as pointed out by recent research [30]. As mentioned in the “Methods” section, the Japanese guidelines at that time and guidelines from other countries [31, 32] recommended RAASis for patients with hypertension and diabetes mellitus, reflecting the results of previous studies that showed the positive effect of decreasing intraglomerular pressure [33] or the antiglycemic effect in a kind of angiotensin-converting enzyme inhibitor [34]. Our results showed that the proportion of patients with diabetes mellitus prescribed RAASi as the first-choice drug was higher in large hospitals than in clinics, as well as in other categories. This result indicated that the importance of RAASis for patients with hypertension and diabetes mellitus was widely acknowledged in specialists in large facilities compared to physicians working in clinics, who generally engage in a general practitioner-like function in Japan. Another possible interpretation is that patients visiting larger hospitals are more likely to have complications such as heart and kidney diseases, of which RAASi had a positive effect on preventing progression. Although diagnosis data contained limited information as discussed above, the difference in the proportion of patients with kidney disease-related diagnosis in large hospitals and clinics (557/3445 vs. 1636/34374) may partially explain the aforementioned association. In addition, recent reviews [35, 36] and guidelines [37] pointed out a factor, named clinical inertia, for preventing treatment-target achievement in hypertension therapy. Clinical inertia is defined as the failure of health care providers to initiate or intensify therapy when indicated [38] and is said to be the consequence of clinicians’ lack of knowledge [36] or patients’ lack of enthusiasm for the management of asymptomatic problems [38]. Hence, it is possible that healthcare providers have little access to updated information concerning hypertension therapy, and patients expect “simple and easy” treatment as well in smaller facilities, resulting in our present data.

In contrast, our results showed that the difference in the proportion of RAASi prescriptions for patients with diabetes was smaller between medium-sized hospitals and clinics than between other categories. It is possible that long-term care hospitals and rehabilitation facilities tended to be categorized into this size strata considering the Japanese healthcare provision situation, and it is also possible that the preference of physicians working in these facilities can be different from those of specialists, but this result is difficult to interpret.

Recently, describing real-world practice by analyzing electronically stored healthcare-related data (e.g., federated electronic medical records, insurance claims database) has become possible [39], and several researchers worldwide have also utilized this data [40, 41]. These researchers noted their novelty in describing practices such as first-line antihypertensive treatment in their countries, but have not yet succeeded in revealing differences among various sizes of facilities. Accordingly, we could not compare our result to those of other countries where healthcare systems do not have a “free-access” attribute to examine the effect of the attribute. Therefore, at this moment, we could not determine whether our situation is desirable or not due to lack of data for comparison, even though we considered our subjective impression from the difference (1.96 vs. 1.69 drugs and 18.9 vs. 12.0%) was small and further role sharing seems to be desirable, considering the increase in healthcare expenditure in Japan. As explained previously, each country has its own healthcare system with particular characteristics related to the gatekeeper function. Hence, our results can be utilized as reference data for the current role sharing situation. In the future, when a sufficient amount of real-world data is collected in each country, researchers will be able to compare their countries’ situation with that of Japan.

### Limitations

First, the database used in this study only contained administrative data, and no blood pressure or chemical examination data were available. Therefore, we could not extract adequate outcomes or comorbidities from the viewpoint of pathophysiology, but could only estimate them from prescription and diagnosis data. Financial incentives for reimbursement and governmental punishment for overcharge may make the prescription data accurate, but the association between prescription claim and patient conditions has not yet been proven. Regarding diagnosis information related to kidney disease, as also mentioned in the discussion section, its sensitivity was reported to be low. Therefore, information related to these strata is limited. Second, we could not distinguish physicians’ intention of prescription other than lowering blood pressure. For instance, we could not omit beta-blocker prescription for heart failure or diuretics for hypervolemia. Owing to this limitation, the mean number of antihypertensive agents increased, and the proportion of refractory hypertension also increased. Finally, the data obtained were those accumulated in only one month, October. We could not take seasonal effects into account, and we might have missed patients whose hypertension was managed but did not visit a healthcare facility during this month. In addition, the consultation interval can be different between large hospitals and clinics due to the difference in background comorbidities, which were not adjusted for in the aforementioned stratification. Therefore, this variation in consultation interval may have also affected our results. Ideally, our investigation would be more accurate when utilizing the whole claims data from the ministry. However, restricting laws and regulations are far stronger if we intend to use the whole data compared to the sampled data that we analyzed in the present study. To effectively examine the current healthcare situation, our method has certain rationality even though some limitations exist.

## Conclusion

The number of prescribed antihypertensive drugs is associated with the size of the prescribing facilities. This result describes the current role sharing situation of hypertension therapy in the Japanese healthcare system, which has a “free-access” attribute and can be utilized as reference data in future studies.

## Supplementary information

Supplementary Figure

Supplementary Table
