# Supplementary figures and images for: Association between the size of healthcare facilities and the intensity of hypertension therapy: a cross-sectional comparison of prescription data from insurance claims data
Source: Hypertens Res. 2020 Sep 15;44(3):337–47. doi: 10.1038/s41440-020-00549-2 (PMC7872892; doi:10.1038/s41440-020-00549-2)

Supplementary Figure


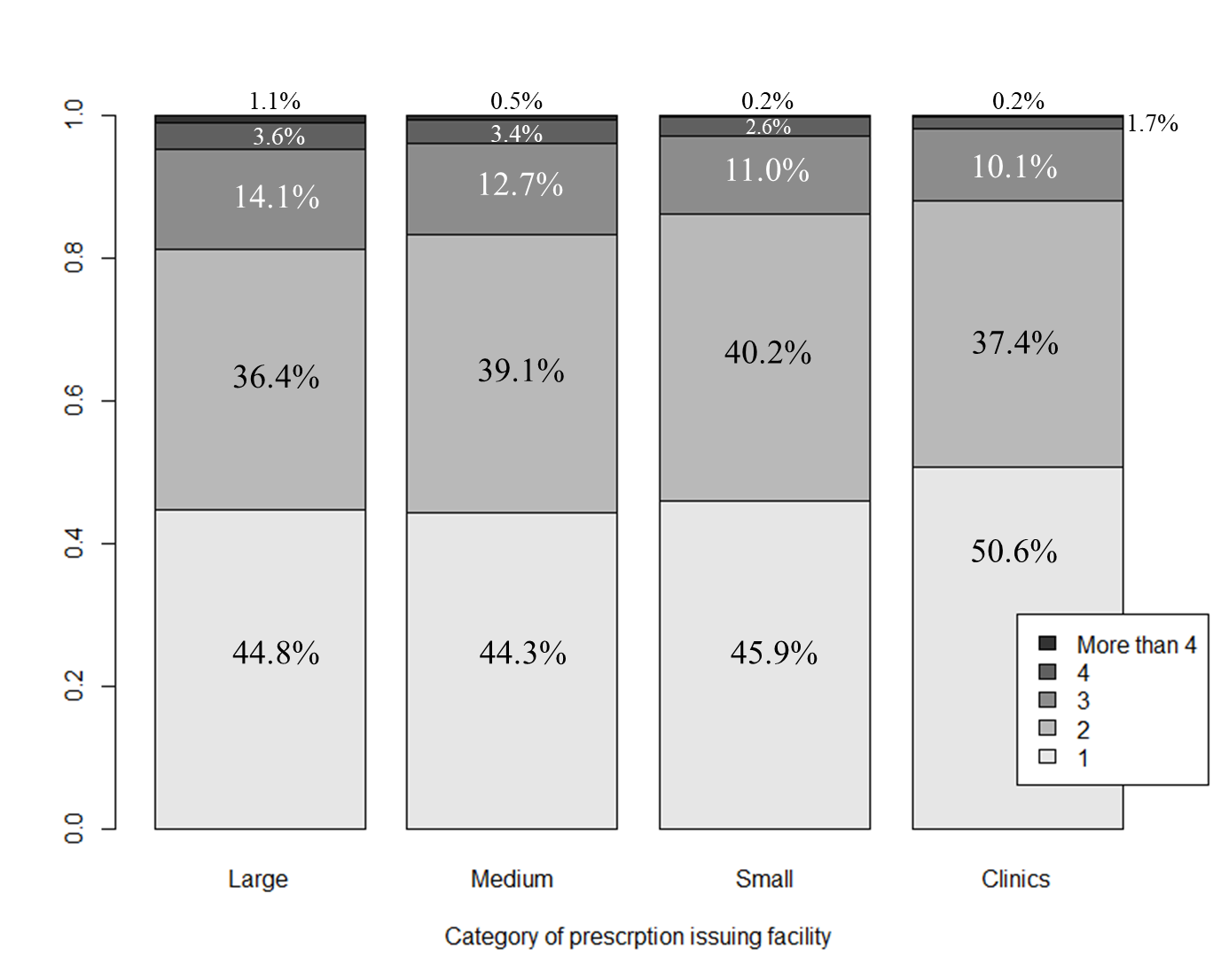

Supplement: Supplementary file 1 — Supplementary Figure [file 41440_2020_549_MOESM1_ESM.docx]
