# Supplementary material for: Association between the size of healthcare facilities and the intensity of hypertension therapy: a cross-sectional comparison of prescription data from insurance claims data
Source: Hypertens Res. 2020 Sep 15;44(3):337–47. doi: 10.1038/s41440-020-00549-2 (PMC7872892; doi:10.1038/s41440-020-00549-2)
Supplement: Supplementary file 2 — Supplementary Table [file 41440_2020_549_MOESM2_ESM.docx]

Supplementary Table. The number of antihypertensive drugs and the proportions of patients prescribed ≥3 classes of antihypertensive drugs with age and sex adjustment (reference group = clinics)

|  |  | Large hospitals | | | Medium sized hospitals | | | Small hospitals | | | Clinics | | |
| --- | --- | --- | --- | --- | --- | --- | --- | --- | --- | --- | --- | --- | --- |
| Subgroups | n (total) | n | Number of  antihypertensive drugs, mean | Proportion of patients prescribed more than 2 classes of antiypertensive drugs | n | Number of  antihypertensive drugs, mean | Proportion of patients prescribed more than 2 classes of antiypertensive drugs | n | Number of  antihypertensive drugs, mean | Proportion of patients prescribed more than 2 classes of antiypertensive drugs | n | Number of  antihypertensive drugs, mean | Proportion of patients prescribed more than 2 classes of antiypertensive drugs |
| Total | 41,939 | 3,445 | 1.95 | 18.8% | 1,797 | 1.85 | 16.6% | 2,323 | 1.79 | 13.8% | 34,374 | 1.69 | 12.0% |
| Age >= 75 | 17,635 | 1,479 | 2.00 | 21.1% | 869 | 1.90 | 18.3% | 1,125 | 1.87 | 15.5% | 14,162 | 1.74 | 13.7% |
| Age < 75 | 24,304 | 1,966 | 1.91 | 17.2% | 928 | 1.82 | 15.3% | 1,198 | 1.73 | 12.6% | 20,212 | 1.66 | 10.7% |
| Co-existence of Diabetes mellitus | 6,069 | 804 | 2.15 | 22.0% | 351 | 2.00 | 21.6% | 385 | 1.86 | 16.6% | 4,529 | 1.81 | 15.3% |
| Without Diabetes mellitus | 35,870 | 2,641 | 1.88 | 17.6% | 1,446 | 1.81 | 15.5% | 1,938 | 1.77 | 13.1% | 29,845 | 1.67 | 11.5% |
| Co-existence of  Dyslipidemia | 16,113 | 1,445 | 2.05 | 22.4% | 703 | 1.87 | 17.6% | 909 | 1.79 | 14.8% | 13,056 | 1.72 | 13.0% |
| Without Dyslipidemia | 25,826 | 2,000 | 1.87 | 15.9% | 1,094 | 1.83 | 15.9% | 1,414 | 1.78 | 13.2% | 21,318 | 1.68 | 11.3% |
| Co-exitence of Kidney disease | 2,507 | 557 | 2.27 | 27.8% | 147 | 2.08 | 30.3% | 167 | 2.07 | 26.7% | 1,636 | 1.96 | 20.7% |
| Without Kidney disease | 39,432 | 2,888 | 1.89 | 17.2% | 1,650 | 1.82 | 15.3% | 2,156 | 1.77 | 13.1% | 32,738 | 1.68 | 11.5% |
